# Methicillin-Resistant *Staphylococcus aureus* Carriage and Risk Factors for Skin Infections, Southwestern Alaska, USA

**DOI:** 10.3201/eid1605.090851

**Published:** 2010-05

**Authors:** A. Michal Stevens, Thomas Hennessy, Henry C. Baggett, Dana Bruden, Debbie Parks, Joseph Klejka

**Affiliations:** Centers for Disease Control and Prevention, Anchorage, Alaska, USA (A.M. Stevens, T. Hennessy, D. Bruden, D. Parks); Centers for Disease Control and Prevention, Atlanta, Georgia, USA (H.C. Baggett); Yukon Kuskokwim Heath Corporation, Bethel, Alaska, USA (J. Klejka)

**Keywords:** Carrier state, skin infections, antimicrobial resistance, bacteria, Staphylococcus aureus, community-acquired infections, MRSA, Alaska, research

## Abstract

Community-acquired methicillin-resistant *Staphylococcus aureus* (CA-MRSA) infections are common in southwestern Alaska. Outbreak strains have been shown to carry the genes for Panton-Valentine leukocidin (PVL). To determine if carriage of PVL-positive CA-MRSA increased the risk for subsequent soft tissue infection, we conducted a retrospective cohort study by reviewing the medical records of 316 persons for 3.6 years after their participation in a MRSA nasal colonization survey. Demographic, nasal carriage, and antimicrobial drug use data were analyzed for association with skin infection risk. Skin infections were more likely to develop in MRSA carriers than in methicillin-susceptible *S. aureus* carriers or noncarriers of *S. aureus* during the first follow-up year, but not in subsequent years. Repeated skin infections were more common among MRSA carriers. In an area where PVL-containing MRSA is prevalent, skin infection risk was increased among MRSA nasal carriers compared with methicillin-susceptible *S. aureus* carriers and noncarriers, but risk differential diminished over time.

## Abstract 2

Skin infection risk was increased among MRSA nasal carriers.

Methicillin-resistant *Staphylococcus aureus* (MRSA) has become a primary cause of skin and soft tissue infections among persons without extensive exposure to healthcare settings. Nasal carriage of *S. aureus* is a known risk factor for these infections (*1*–*3*) and a common reservoir during skin and soft tissue infection outbreaks (*4*–*6*). Such outbreaks have occurred in community (*7*–*10*) settings, e.g., athletic team facilities (*11*–*16*), correctional facilities (*13*,*17*), and military basic training camps (*18*,*19*). Risk factors found in these community settings are frequent skin-to-skin contact (*11*,*12*,*14*,*15*), sharing of personal items without frequent cleaning (*11*–*14*), and MRSA carriage (*18*,*20*). Nasal colonization is also a risk factor for infections in hospital settings (*4*–*6*,*21*–*23*) and long-term care facilities (*24*,*25*). However, all of these previous studies have used a case–control study design, making it impossible to determine if MRSA carriage preceded infection.

Little is known about the subsequent risk for skin and soft tissue infections among persons colonized with MRSA. This lack of information becomes a question of clinical significance because increasing numbers of MRSA case-cluster investigations include nasal colonization studies that identify persons as MRSA-colonized. Clinicians and patients are left to consider whether interventions such as decolonization, continued monitoring, or restrictions in occupational activities are indicated. The notable absence of data regarding subsequent risk for illness among MRSA-colonized persons in community settings does little to inform these treatment decisions.

In southwestern Alaska, a dramatic increase in the number of skin infections led to an investigation by the Centers for Disease Control and Prevention and the Yukon Kuskokwim Health Corporation in 2000. MRSA exhibiting the type IV staphylococcal cassette chromosome *mec* gene had become the predominant community strain in that region, accounting for 100% of *S. aureus* isolates from skin infections (*7*,*8*). Also, the USA400 strain is the predominant strain of CA-MRSA in this area, whereas USA300 is the predominant strain in most other areas of the United States (*8*). In that initial investigation, we conducted a case–control study in 1 village in Alaska to assess risk factors for MRSA skin infections and evaluated nasal carriage among case–control participants and their household members (*1*). The present study is a follow-up assessment of participants in the prior nasal colonization survey. Our goal was to assess the risk for subsequent skin infections among persons whose nares cultures were colonized with MRSA (carriers) compared with those colonized with methicillin-susceptible *S.* *aureus* (MSSA), or those whose nares cultures were negative for *S. aureus* (non–*S. aureus* carriers).

## Methods

This retrospective cohort study included persons who had been enrolled in a case–control study conducted in September 2000, which included anterior nares swab cultures for *S. aureus* obtained by using standard methods (*1*). The 316 study participants included 32 persons with a history of a culture-confirmed *S. aureus* skin infection (furunculosis or cellulitis) in the data collection period, 90 persons with no skin infection history in the year before the case–control study, and 194 household members of case- and control-patients. All persons were residents of 1 southwest Alaska village (population 713, accessible only by river or airplane) (*26*). Healthcare is provided by 1 integrated system, which includes a primary care clinic in the village and 1 regional hospital. The study was approved by the Institutional Review Board of the Centers for Disease Control and Prevention, area review boards in Alaska, and the local tribal health authority. A waiver of informed consent was obtained that enabled use of data from existing sources after delinking from individual identifiers.

### Data Collection

We reviewed the medical records of all 316 persons to determine the total clinic or hospital visits by patients with skin infections or those for which an antimicrobial drug was prescribed during September 20, 2000–May 2, 2004 (3.6 years). Each participant’s age, sex, *S. aureus* carriage status at the beginning of the study, and whether he or she lived with a MRSA carrier were recorded. We defined an antimicrobial drug course as a prescription for any oral or parenteral antimicrobial agent. Prescriptions involving simultaneous administration of >1 antimicrobial drug were counted as 1 course, as were changes in antimicrobial treatments for the same illness course that may have resulted from a participant’s inadequate response to empiric therapy. For continuous prophylactic antimicrobial drugs, each month was counted as 1 course of treatment. Topical antimicrobial drugs were not counted.

Skin infections were defined as furuncles/abscesses, cellulitis, folliculitis, or deep wound infections as documented by the clinical provider. For each patient, we recorded visit date, diagnosis, anatomic site of infection, pathogen (if available), and consequent antimicrobial drug therapy. Visits for impetigo, scabies, and dermatitis were not included in the analysis. We considered skin infections to be distinct episodes if they occurred in the same anatomic site >6 months apart, and in different anatomic sites if they occurred >2 months apart. Only the first visit for treatment of a skin infection episode was recorded if there were multiple visits for the same episode. Nasal colonization was assessed only at the onset of the study. Isolates from skin infections of study participants were not available for comparison with nasal colonization specimens.

### Statistical Analysis

Univariate analyses were performed to compare demographic and clinical characteristics among 3 carriage groups (MRSA, MSSA, and non–*S. aureus* infections). Demographic characteristics included age, sex, and *S. aureus* colonization status of household members. Clinical characteristics included the type and anatomic site of the skin infection, identified pathogens, and current history of antimicrobial drug treatment. Analyses were performed by using SAS version 8 software (SAS Institute, Inc., Cary, NC, USA). Univariate comparisons of categorical and continuous variables were conducted by using the χ^2^ and Kruskal-Wallis tests, respectively. We assessed confounding of demographic characteristics through use of the Cochran-Mantel-Haenszel test. The Kaplan-Meier method was used to determine risk for first skin infection over time for each of the 3 groups. The log-rank statistic was used to compare survival (time without a skin infection) between carriage groups. Persons were censored at the time of death (n = 5) or at the time of last contact with the healthcare system for those who were known to have moved from the village (n = 3). We assessed confounding of other variables with a stratified nonparametric survival test (*27*).

## Results

### Study Population

Of the 316 participants, 41 (13%) were colonized with MRSA at the beginning of the study period (September 20, 2000); 85 (26.9%) were colonized with MSSA, and 190 (60.1%) were not colonized with *S. aureus*. Nasal carriage findings are described in more detail in a report of the prior case–control study (*1*). For the purposes of this study, 10 (24.4%) of those colonized with MRSA had been included as case-patients with a history of skin infections in the prior case–control study, 3 (7.3%) were control-patients with no history of skin infections in the previous year, and 28 (68.3%) were household members of case- or control-patients whose skin infection history before the study period was not obtained. Of those colonized with MSSA, 2 participants were case-patients, 28 were control-patients, and 55 were household members. Of the noncarriers, 20 were case-patients, 59 were control-patients, and 111 were household member participants. MRSA carriers were more likely to have a household member who was also a MRSA carrier (78%) compared with MSSA carriers (29%) and non–*S. aureus* carriers (17%) (p<0.0001). *S. aureus* carriers were, on average, younger (median 14 years) than non–*S. aureus* carriers (median 22 years, p = 0.02) (Table 1). However, MRSA carriers (median 13 years) and MSSA carriers (median 16 years) were of similar ages (p = 0.46).

**Table 1:** Characterization of study participants by *Staphylococcus aureus* carriage group, Alaska, 2000*

Characteristic	MRSA carriers, n = 41	MSSA carriers, n = 85	Non–*S. aureus* carriers, n = 190	Total, n = 316	p value†
Male sex	23 (56.1)	40 (47.1)	94 (49.5)	157 (49.7)	0.6
Median age, y (range)	13 (0–77)	16 (1–73)	22 (0–85)	18 (0–85)	
Age group, y					
<5	10 (24.4)	17 (20.0)	29 (15.3)	56 (17.7)	0.0456
5–19	15 (36.6)	35 (41.2)	57 (30.0)	107 (33.9)	
20–39	9 (22.0)	20 (23.5)	60 (31.6)	89 (28.2)	
40–64	6 (14.6)	9 (10.6)	32 (16.8)	47 (14.9)	
>64	1 (2.4)	4 (4.7)	12 (6.3)	17 (5.4)	
Household member nasal carriage status					
>1 MRSA+	32 (78.0)	25 (29.4)	32 (16.8)	89 (28.2)	<0.0001

*Values are no. (%) except as indicated. MRSA, methicillin-resistant *S. aureus*; MSSA, methicillin-susceptible *S. aureus*. †χ^2^ tests were used to detect differences in carriage groups by sex and household member status; nonparametric 1-way analysis of variance software (SAS Institute, Cary, NC, USA) was used to detect differences in carriage groups by age.

### Risk for Skin Infection

During the first year after the carriage study, >1 skin infections developed in 107 (33.9%) participants. Skin infections were more likely to develop in MRSA carriers (23/41 [56.1%]) compared with MSSA carriers (27/85 [31.8%], relative risk [RR] 1.76) and non–*S. aureus* carriers (57/190 [30.0%], RR 1.87, p = 0.005). For persons who did not have a skin infection diagnosed in the first year, the risk for developing a skin infection in the 2 to 3.6-year period did not differ significantly by carrier status: 5/18 (27.8%) of MRSA carriers, 20/58 (34.5%) of MSSA carriers, and 59/133 (44.4%) of noncarriers (p = 0.23). Over the entire 3.6-year follow-up period, >1 skin infections developed in a higher proportion of MRSA carriers (28/41 [68.3%]) than in MSSA carriers (47/85 [55.3%]) and non–*S. aureus* carriers (116/190 [61.1%]), but these differences were not statistically significant (p = 0.36). Rates for skin infections for the first 3 years of follow-up are shown in Table 2.

**Table 2:** *Staphylococcus aureus* skin infections among study participants, by year, Alaska, 2000–2004*

Skin infection outcome	MRSA carriers, n = 41	MSSA carriers, n = 85	Non–*S. aureus* carriers, n = 190	p value	
				MRSA vs. non–*S. aureus*	MSSA vs. non–*S. aureus*
Skin infection rate, %					
Year 1	56	32	30	0.001	0.77
Year 2	37	26	30	0.42	0.50
Year 3	32	21	21	0.15	0.98
Cumulative % with >1 skin infection†					
Year 1	56	32	30	0.001	0.69
Year 2	61	41	48	0.02	0.48
Year 3	66	52	58	0.07	0.50
1st year of follow-up					
Mean no. skin infections‡	0.7	0.4	0.4	0.001	0.73
No. (%) with >2 infections§	6 (15)	7 (8)	13 (7)	0.10	0.68
Entire follow-up period, 3.6 y					
Mean no. skin infections‡	1.9	1.2	1.1	0.03	0.60
No. (%) with >3 infections§	13 (32)	13 (15)	24 (13)	0.02	0.72

*MRSA, methicillin-resistant *Staphylococcus aureus*; MSSA, methicillin-susceptible *S. aureus*. Skin infection rate per 100 persons in each of the 3 years; 2-sample Poisson test was used to compare skin infection rates. †Comparisons made by use of Log-Rank statistic in Kaplan-Meier estimation of survival curve. ‡Comparisons made by use of Kruskal-Wallis statistic. §Chi-square test used for calculation of p value.

The median age of persons in which skin infections developed in the first year was 17 years and was similar for each carriage group (p = 0.50). Among persons in which skin infections developed during the full study period, the median age was similar for MRSA carriers (median 16.5 years) and MSSA carriers (median 14 years, p = 0.7), but was higher for non–*S. aureus* carriers (median 22 years, p = 0.06 vs. MRSA carriers).

The number of skin infections in the first year was higher for MRSA carriers (mean 0.7) than for either MSSA carriers (mean 0.4) or non–*S. aureus* carriers (mean 0.4, Table 2). For the entire follow-up period, MRSA carriers had a mean of 1.9 skin infections per person, which exceeded that of MSSA carriers (mean 1.2, p = 0.04) and the non–*S. aureus* carriers (mean 1.1, p = 0.02). No difference was detected in the numbers of skin infections by sex and carriage group in either analysis of the first year (p>0.3) or the full study period (p>0.9).

Among MRSA-colonized persons, skin infection risk did not differ by the colonization status of household members (p>0.22). However, among non–MRSA-colonized persons, skin infection risk in the first year was higher for those with a MRSA–colonized household member (23/57 [40%]) compared with those without a MRSA−colonized household member (61/208 [24%], RR 1.4, 95% confidence interval [CI] 1.0–2.1, p = 0.07); this difference did not persist when considering the entire follow-up period (p = 0.4). After adjusting for household member MRSA colonization, the RR for developing a skin infection in the first year was 1.6 (95% CI 1.1–2.4) when comparing MRSA carriers versus non–*S. aureus* carriers.

By using survival analysis, we found that after 1 year MRSA carriers were more likely than non–*S. aureus* carriers to have had a skin infection (RR 1.9, 95% CI 1.3–2.6, p = 0.001; Table 2). After 1 year, no difference was detected in the occurrence of a first skin infection between MSSA carriers and non–*S. aureus* carriers (p = 0.69). After 2 years, an increased risk remained between MRSA and non–*S. aureus* carriers (p = 0.02), but after 3 years this difference was no longer significant (Table 2). We stratified by gender, age, and household member colonization status; the results remained unchanged. The estimated length of time for 50% of the MRSA carriage group to have developed a skin infection was 289 days (0.8 years), 1,049 days (2.9 years) for MSSA carriers, and 804 days (2.2 years) for non–*S. aureus* carriers (Figure).

**Figure:**
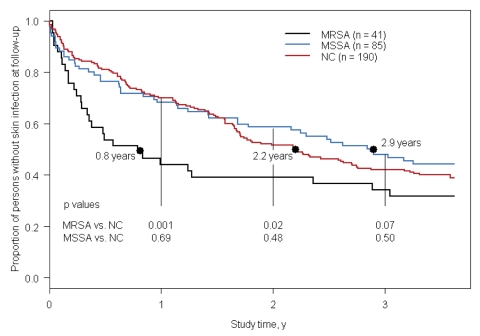
Kaplan-Meier survival curve of time until first skin infection among methicillin-resistant *Staphylococcus aureus* (MRSA), methicillin-susceptible *S. aureus* (MSSA), and non–*S. aureus* carriers (NC). Black dots and associated text show the median time to first skin infection for each of the 3 groups.

### Skin Infection Characterization

A total of 391 skin infections occurred during the 3.6-year follow-up. Of these, 246 (62.9%) presented as a single furuncle; 85 (21.7%) were multiple furuncles; 50 (12.8%) had evidence of cellulitis; 4 (1.0%) were diagnosed as folliculitis; and 6 (1.5%) were deep abscesses. One hundred thirteen (45.9%) single boils occurred in the buttocks/low back/thigh area; 35 (41.2%) multiple boil infections occurred on the buttocks/low back/thigh area. Thirty-three (66.0%) of the cellulitis infections occurred on the extremities.

For all skin infections on which antimicrobial drug data were available, 264 of 382 (69.1%) patients were prescribed antimicrobial drugs. We found no difference in the mean number of antimicrobial drug treatment prescribed over the study period to *S. aureus* carriers compared with non–*S. aureus* carriers (12.0 vs. 10.4, respectively; p = 0.12) or for MRSA carriers (mean 13.6) as compared with MSSA carriers (mean 11.3, p = 0.17).

During the entire course of follow-up, 79/383 (21%) of infections (7 with an unknown culture status) were cultured. The proportion of skin infections cultured was similar for MRSA carriers (22%), MSSA carriers (22%), and non–*S. aureus* carriers (20%). In the 3.6 year follow-up period, 70 (89%) skin infections cultured were MRSA, 6 (8%) were MSSA, and 3 (4%) were other pathogens. Over the entire study period, 12/41 (29%) of MRSA carriers had a MRSA confirmed skin infection, compared with 12/85 (14%) of MSSA carriers and 30/190 (16%) of non–*S. aureus* carriers (p = 0.05 for MRSA vs. MSSA carriers, p = 0.004 for MRSA vs. non–*S. aureus* carriers, and p = 0.72 for MSSA vs. non–*S. aureus* carriers).

## Discussion

In this study of a rural village in southwestern Alaska where MRSA was responsible for 86% of skin infections, we recruited a cohort of participants from a community setting to determine risk factors for the development of skin infections. We found nasal MRSA carriage to be a significant risk factor for skin infections in the first year when compared with MSSA and non–*S. aureus* carriers. More skin infection episodes also developed in these MRSA carriers than other carriage groups in the first year and entire 3.6-year follow-up period. We note that the risk for skin infections among MRSA carriers decreased with time but not for MSSA or non–*S. aureus* carriers; rates did not differ significantly between the 3 groups by the end of the study period. MRSA was the cause of 90% of skin infections in the follow-up period, with a similar proportion of cultures obtained among the carriage groups. Notably, skin infection took longer to develop in MSSA carriers, which may suggest that MSSA carriage provides some protection against MRSA infection. The strengths of this study are the long follow-up period, the single healthcare system that enabled capture of all clinic and hospital visits, and the location (an isolated community, which had recently experienced an outbreak of MRSA skin infections).

Having a household member who was a MRSA carrier was associated with an increased risk for skin infection in the first follow-up year for MSSA carriers and non–*S. aureus* carriers but not for MRSA carriers. Studies by Boubaker et al. (*28*) and Osterlund et al. (*9*) have shown that household members of MRSA–colonized persons are at increased risk for becoming colonized. Our data support the hypothesis that transmission of MRSA from carriers to non-colonized household members is a risk factor for disease acquisition. Transmission of MRSA from household carriers during the follow-up period may also explain the continued risk for skin infections observed among MSSA and non–*S. aureus* carriers throughout the study. In contrast, the risk for skin infection among MRSA carriers was greatest in the first follow-up year but decreased thereafter, possibly indicating acquisition of immunity in this group. It is crucial to note that after the initial assessment the nasal carriage status of participants is unknown.

In the case–control study that preceded this investigation, antimicrobial drug use in the 12 months preceding the MRSA outbreak was associated with an increased risk for MRSA infection (*1*). However, in this study antimicrobial drug use did not differ among the 3 carriage groups and thus was not associated with subsequent risk for disease from MRSA. The small sample size of cultured skin infections and changes in clinical guidelines limit detection of any association. Antimicrobial drug use may still be a risk factor for MRSA infection in this community, although it was not demonstrated in this cohort. Alternatively, this finding may indicate a decreased role for antimicrobial drugs as a risk factor for MRSA infection once MRSA is established as a common colonizing organism in a community.

Our study population experienced a high incidence of skin infections compared with those in other published reports. In the first follow-up year, we found that 56% of MRSA carriers, 32% of MSSA carriers, and 30% of non–*S. aureus* carriers developed skin infections. A similar study by Muder et al. in a long-term care facility showed infection rates of 25%, 4%, and 4% for the same carriage groups, respectively, but patients were only followed while in the hospital and median duration of follow-up was <1 year for the carriage groups (*24*). Another prospective study of soldiers by Ellis et al. found that 38% of MRSA carriers, 3% of MSSA carriers, and 2% of non–*S. aureus* carriers developed subsequent skin infections (*18*). The higher incidence of skin infections may have been due, in part, to the absence of piped in-home water and wastewater service in this village. Lack of in-home running water has been associated with increased rates of skin and respiratory tract infections in rural Alaska, presumably due to decreased opportunities for hand and body hygiene. When household water must be carried into the home in buckets, residents may not wash their hands or bathe as often as they would if they had water available by turning a tap (*29*). The high skin infection rates could also be due to MRSA-colonized biofilms in saunas; 49% of saunas tested were positive for outbreak-strain MRSA. Sauna use >2 hours per week was reported by 68% of participants (*1*).

This study had several limitations. Persons with a history of frequent skin infections before the follow-up period may have been more likely to develop skin infections during the chart review period. However, we were not able to control for the potential confounder of past skin infections because of the limited dataset available on the cohort. Selective pressure for CA-MRSA carriage may have diminished during the follow-up period, because new antimicrobial drug treatment guidelines were implemented to reduce overtreatment with broad-spectrum antimicrobial drugs and first-generation cephalosporins as the outbreak progressed. Another limitation is that *S. aureus* nasal carriage status was assigned based on cultures performed at the beginning of the study period but colonization status was not assessed further; nasal carriage of *S. aureus* is known to be naturally transient in many carriers, or may also have been affected by use of antimicrobial drugs. Therefore, the effect of duration of carriage or crossover from 1 study group to another could not be determined. Study participants could have moved or been lost to follow-up, therefore these data represent minimal incidence estimates for the population. Another limitation is that behavioral data were not available for known risk factors for MRSA skin infections, such as sauna use or skin contact. MRSA carriage can occur in other body sites, such as the groin or axillae; our nasal carriage survey may have underestimated actual MRSA carriage.

Our study supports the hypothesis that nasal carriage of MRSA is a risk factor for skin infections, and that the risk may decrease over time relative to MSSA carriers and non–*S. aureus* carriers. We found that MRSA carriage among household members increased the risk for skin infection among non–MRSA carriers. This information may be useful for education of persons identified as MRSA carriers or with MRSA–colonized household members to reinforce the value of hand hygiene practices and other measures that have been recommended to prevent the spread of MRSA within households. Further study of MRSA transmission in community settings is needed along with interventions that are designed to minimize pathogen transmission to close contacts and household members.
